# Safe Motherhood Case Studies: Learning from South Asia

**DOI:** 10.3329/jhpn.v27i2.3322

**Published:** 2009-04

**Authors:** Jennifer Amery

**Affiliations:** Senior Health Adviser, South Asia Region, DFID India, UK Department for International Development (DFID), B28 Tara Crescent, Qutab Institutional Area, New Delhi 110 016, India

Generations of our children and grandchildren may rightly look back and wonder how so many women were allowed to die from preventable conditions in pregnancy and childbirth in the first decades of the 21^st^ century, when so much effort was being invested in biomedical, space and weapons technologies.

The case studies published in this issue of the Journal grew from discussions between concerned individuals in 2004 about the need to share lessons from low-income contexts in South Asia, specifically India, Pakistan, and Bangladesh, where progress had been made to reduce the number of maternal deaths, focusing on the why and how. The aim is for the lessons from these countries and the good practice developed in some of these remarkable initiatives to inform and, thus, contribute to more rapid progress elsewhere in South Asia and beyond.

There are at least five key challenges to reducing the number of maternal and neonatal deaths in South Asia. The first challenge is what types of services are affordable and feasible in a given context. Enough is known about which women are dying and why (1) and what works in terms of the technical interventions needed (2), to advocate scaling up service-delivery. However, for decision-makers, managers, and committed clinicians in settings of weak or absent primary healthcare services, referral systems, emergency transport, and emergency obstetric care, with an unskilled or semi-skilled health workforce for normal and complicated deliveries, it is not always clear where to start, especially where demand for maternity services is low and most maternal deaths are invisible to the health services, authorities, and the media. There is no single right model of service-delivery. In the huge and varied contexts of the large countries included here, we learn just how much context does matter. In these case studies, we are looking for patterns among the solutions to inform us, rather than ‘models’ to be rolled out or replicated. In each of these case studies, bold steps were taken, starting with what was available.

A second challenge is how to develop, retain, and reward an appropriately-skilled and motivated workforce. In South Asia, this is perhaps the key constraint to progress and the most politically tricky. There is good evidence emerging on how to go to scale with professional care (3). Midwives are one necessary key to success in reducing the number of maternal deaths in many developed and developing countries but are too often seen as a threat to the medical establishment. In South Asia, they are far too a few in number and have low status and little voice. In particular, skilled midwives working in teams to provide services round-the-clock can substantially increase access to safe delivery and basic emergency obstetric care. A second key is the obstetrician/anaesthetist team. Given the continued lack of such teams throughout rural South Asia, there is a welcome focus, for example in India and Bangladesh, on raising skills of non-specialist doctors to do caesarean sections and to give anaesthesia. However, the gap is overwhelming, and there is perversely a risk of over-medicalization and escalating caesarean-section rates in some places and exploitation of poor people. There is a need to create dynamic teams for maternity care, with a significant role for midwives, including in the delivery of basic emergency obstetric care. Horizontal cross-professional training, followed by employment of professionals as part of a team, who are socialized as a team, and possibly rewarded as a team, needs to be further explored.

Getting the right mix of skilled health workers where they are most needed is perhaps the most pressing task. In a public sector where there is no rule-based system or transparency in the recruitment, posting, or transfer of health workers and where political patronage is maintained precisely by this lack of transparency and accountability, this seems a herculean task. However, these case studies show that some committed decision-makers in the public system have found ways to make progress. Elsewhere, public funds have been used for harnessing the private for-profit sector to deliver maternity services for poor women.

The third challenge is access to maternity services for all women, regardless of their caste, ethnicity, religion, economic or social status. There are huge differences in access to maternity services between the highest and the lowest wealth quintiles (4,5), and poor rural women often have no chance of reaching emergency life-saving services when they need them. Some women are excluded from services even if they live a short distance away from a health facility. Inquiries and audits of maternal and perinatal deaths at the subdistrict level in India have documented details of women sent from facility to facility, only to return home to die. The underlying social norms and the distribution of power must be challenged if women are to stop dying in pregnancy and childbirth (6). This is not a task just for the health services but health workers also must be part of the solution, and not part of the problem, and treat all women according to their needs.

Fourth, for all women to access maternity and other health services, the service providers must be accountable to the community and to the users. As with the challenges of an appropriate workforce and of equitable access to health services, this challenge goes beyond the health sector (7). Recent demand-side financing initiatives in India and Bangladesh, whereby women are given a payment if they come to a health facility for the delivery, may send a message that the state is now attaching importance to maternity services. They may help raise the visibility of maternal deaths and bring health services authorities to account, particularly if they are monitored by a committee that oversees citizens' concerns. For too long womens' expectations of what health services can offer them have been too far low, and there have been no systems to hear grievances or provide redress for failings of health service providers.

Fifth, there is the challenge of obtaining and using information to improve maternal health at the community level and district and national levels, to know what progress is being made. Linked to this is the need to expand the evidence-base of how to make progress in particular settings. These case studies offer a contribution to that evidence-base of the operational approaches that have made a difference. Some are at an early stage of implementation, and further analysis is needed over time. Some parts of South Asia may leap-frog to 21^st^ century communications technology, with telemedicine brought to the village. However, many hundreds of millions of poor women will continue to rely on committed local health workers to save their lives in pregnancy and childbirth, and it is vital to know how best to deliver services to ensure that their lives and those of their babies will be saved.

These case studies do not look explicitly at the costs of different approaches. That is a different but important task, particularly for expanding services to the scale needed in the large populations of South Asia.

I am delighted that this work has come to publication, and that on the way, the key people involved in these case studies have met on several occasions to share experiences across the three countries of India, Pakistan, and Bangladesh. I sincerely hope that the links made and friendships forged will further contribute to the reduction of maternal and neonatal deaths in the region.
